# DL-Methionyl–DL-Methionine/DL-Methionine Supplementation Alleviated the Adverse Effects of Dietary Low Fishmeal Levels on Growth and Intestinal Health of *Micropterus salmoides*

**DOI:** 10.3390/antiox13030359

**Published:** 2024-03-18

**Authors:** Heng Yu, Karthik Masagounder, Hualiang Liang, Xianping Ge, Dongyu Huang, Chunyu Xue, Mingchun Ren, Juyun He

**Affiliations:** 1 Wuxi Fisheries College, Nanjing Agricultural University, Wuxi 214081, China; 2 Evonik Operations GmbH, Hanau-Wolfgang, 63457 Hanau, Germany; 3 Key Laboratory of Integrated Rice-Fish Farming Ecology, Ministry of Agriculture and Rural Affairs, Freshwater Fisheries Research Center, Chinese Academy of Fishery Sciences, Wuxi 214081, China; 4 School of Pharmaceutical Sciences, Guangzhou Medical University, Guangzhou 511436, China

**Keywords:** *Micropterus salmoides* (*M. salmoides*), Met-Met, intestinal microbiota, antioxidant capacity, anti-inflammatory

## Abstract

DL-methionyl–DL-methionine (AQUAVI® Met-Met) (Met-Met) (0.10%, 0.20%, 0.30%, and 0.40%) or DL-methionine (DL-Met) (0.10%, 0.20%, 0.30%, and 0.40%) were added to a low-fishmeal diet in an attempt to reduce fishmeal in the diet of *Micropterus salmoides* (*M. salmoides*). The fish were randomly allocated into ten experimental groups (*n* = 100), each with 4 replicates of 25 fish (16.39 ± 0.01 g) each. Compared to 25% FM, 0.40% of DL-Met and 0.10% of Met-Met promoted growth, and 0.10% of Met-Met decreased FCR. Compared to 25% FM, the supplementation of Met-Met or DL-Met improved the intestinal antioxidant capacity by upregulating the NF-E2-related factor 2-mediated antioxidant factors and enzyme activities and nuclear factor kappa-B-mediated anti-inflammatory factors while downregulating the pro-inflammatory factors, thereby exerting anti-inflammatory effects. Moreover, 0.10% of the Met-Met diet affected the Firmicutes-to-Bacteroidota ratio, increased the levels of Proteobacteria, changed the composition of intestinal flora (*Roseburia*, *Lachnospiraceae_NK4A136_group*, and *unclassified_Oscillospiraceae*), and enhanced intestinal dominant bacteria (*Caldicoprobacter*, *Pseudogracilibacillus*, and *Parasutterella*), leading to improved gut health. In summary, the supplementation of DL-Met or Met-Met alleviated the adverse effect of fishmeal reduction (from 40 to 25%) on the growth performance and intestinal health of *M. salmoides*.

## 1. Introduction

Nutritional balance is closely related to human health, and fish is crucial for the human diet due to its rich nutritional content. Fish meat is a source of rich and high-quality protein that is more accessible and affordable than other animal proteins [1,2]. Since the 1950s, aquaculture production increased globally, while fishery production remained consistent since the early 1990s [3]. Moreover, the production of aquaculture for human consumption exceeded that of the fisheries in 2016 [3]. Aquaculture plays an important role in world food security by providing aquatic protein [4]. According to a report by FAO [5], the production of freshwater fish is at least five times that of marine fish, demonstrating the important role of freshwater fish farming in food security. *Micropterus salmoides* (*M. salmoides*) is a native to North America and was imported into China for farming as food fish. *M. salmoides* has fast growth, strong adaptability, and delicious meat [6]; therefore, its production increased from 243,196 to 802,486 tons during the last decade in China [7]. However, the increased production necessitates sufficient feed and raw materials for culturing *M. salmoides*. Fishmeal (FM) is one of the most important, expensive, and high-quality sources of protein due to its good palatability, amino acid balance, and high nutritional value [8]. However, FM production and its price are projected to increase by 1% and 30%, respectively, by the year 2030 [9], which may impede the sustainable development of the aquaculture industry. As a carnivorous fish, *M. salmoides* requires 40–50% more FM than herbivorous and omnivorous fish [10]. Therefore, reducing the proportion of FM in *M. salmoides* feed is crucial to overcoming the FM production challenge, thereby promoting the sustainable farming of *M. salmoides*.

To reduce the FM content, animal- and plant-based protein sources are used as important ingredients in aquatic animal feed. However, due to an imbalance of essential amino acids (EAAs), their extensive use results in several adverse effects [11,12,13]. Therefore, it is important to balance the essential nutrients, including EAAs, in the feed to replace FM with animal- and plant-based proteins without affecting the production performance of the fish. A potential alternate protein source is poultry by-products, as they have similar protein contents, reasonable prices, and a stable supply [14]. However, one of the limiting amino acids in poultry by-product feed is methionine [15], which should be included in aquatic animal feed to reduce the adverse impact on growth, antioxidant capacity, and immune function resulting from reduced FM content [16].

Supplementation with EAAs, particularly methionine (Met), was considered an effective way of replacing FM with animal- and plant-based proteins in aquatic feeds [12,17,18]. Methionine products are commercially available in various forms, such as DL-methionine (DL-Met) or Met-Met. Met-Met has an advantage over DL-Met in that it is insoluble in water and absorbed easily [19,20]. Guo et al. found that Met-Met supplementation could improve the growth performance and antioxidant capacity of Nile tilapia (*Oreochromis niloticus*) [20]. Mamauag et al. reported similar utilization effects of DL-Met and Met-Met by Red Sea Bream (*Pagrus major*) larvae and juveniles [21]. Met-Met is mainly used to supplement the feed of *Litopenaeus vannamei* (*L. vannamei*) with a higher availability than DL-Met [19]. Dietary supplementation with 0.34% of Met-Met could reduce the FM content from 18 to 6% without any adverse effect on the performance of *L. vannamei* [22]. Moreover, Met-Met supplementation ameliorates the negative effects on growth caused by limiting Met content in low-FM diets and effectively improves the immune and antioxidant capacity of *L. vannamei* [23]. However, considering the differences between shrimp and fish, the present study investigated the effects of Met-Met supplementation on the growth, intestinal antioxidant capacity, immunity, and microbiota of *M. salmoides*. The outcomes would facilitate the further evaluation of the application of Met-Met in different species, thereby limiting the FM content to promote the sustainable development of *M. salmoides*.

## 2. Materials and Methods

### 2.1. Experimental Design

*M. salmoides* were initially placed in floating net cages in a pond for two weeks for environmental acclimation. Thereafter, the fish were randomly allocated into ten experimental groups (*n* = 100), each with 4 replicates of 25 fish (16.39 ± 0.01 g) each. Subsequently, *M. salmoides* was fed to satiety twice daily (07:30 and 17:30 h) for 10 weeks. The key indicators of water quality monitoring during the culture cycle are listed in Table 1.

### 2.2. Experimental Diets and Feeding Plan

Met-Met and DL-Met were procured from Evonik Operations GmbH (Hanau, Germany). The commercial product (AQUAVI^®^ Met-Met) is a mixture of four different methionine stereoisomers (LD-Met-Met, DL-Met-Met, LL-Met-Met, and DD-Met-Met) [19]. The following dietary formulations were used: (1) 40% FM, 25% FM, and 25% FM supplemented with varying levels of Met-Met (0.10%, 0.20%, 0.30%, and 0.40%) or DL-Met (0.10%, 0.20%, 0.30%, and 0.40%). The ingredient and proximate analysis of the experimental diets are shown in Table 2. Amino acid content in diets and Met-Met and DL-Met levels after supplementation are shown in Table 3. The raw protein materials were crushed using a pulverizer and re-screened through a 60-mesh screen. The resultant protein ingredients were mixed sequentially according to the principle of gradual mixing with water and oil supply. Thereafter, an F-26 (II)-type granulator was used to obtain feed granules. After drying, the prepared feed was stored at −20 °C for further use.

### 2.3. Sampling

The fish were fasted for 24 h before sample collection. A total of 8 fish per group were selected to evaluate the whole-body composition. In addition, two extra fish were randomly selected from each net cage. In other words, the intestinal antioxidant indices and gene expression levels were tested using 8 fish from each group. Furthermore, intestinal tissue samples from three randomly selected fish from each of the 40% FM (HF), 25% FM (LF), and 25% FM supplemented with 0.10% Met-Met groups (LFM) were used to analyze intestinal microbes. The collected samples were stored at −80 °C for further analysis.

### 2.4. The Nutrient Composition and Intestinal Antioxidant Parameter Assays

The experimental assay methods with details of manufacturers or assay kits utilized for the analyses of serum, intestinal antioxidant parameters, whole fish, and diet composition are elaborated in Table 4.

### 2.5. Microbial DNA Extraction and 16S rDNA Sequencing

The intestinal tissues from three *M. salmoides* fed with HF, LF, and LFM diets were used for microbiological analysis. The constructed libraries were screened and sequenced on Illumina NovaSeq 6000. Effective Reads were obtained by quality filtering, double-ended sequence splicing, and chimera removal. USEARCH software (version 10.0) [25] was used to cluster Reads and obtain OTUs with 97.0% similarity.

### 2.6. 16S rDNA Sequencing Data Analysis

The α- and β-diversities were analyzed using QIIME2, and the diversity index was tested by the independent sample *t*-test in SPSS 20. The featured sequences were then classified and labeled by a plain Bayesian classifier, according to the Silv.138 database. The community structure of samples at the taxonomic level (phylum, class, order, family, genus, and species) was calculated by QIIME2 (2020.6) software to generate abundance at different taxonomic levels and mapped using R-language means.

### 2.7. Quantitative Real-Time PCR Detection

The intestinal tissues of *M. salmoides* stored at −80 °C were transferred to dry ice, followed by RNA extraction using the standard reagents by Vazyme (Nanjing, China) according to the manufacturer’s instructions. The RNA quantification and qualitative analysis for further experiments utilized a spectrophotometer (NanoDrop 2000, Thermo Fisher Scientific) (Waltham, MA, USA). HiScript^®^ II One Step qRT-PCR SYBR Green Kit (Vazyme, Nanjing, China) was used to configure the system and detect gene expression levels. The primer sequences are shown in Table 5. Beta-actin (β-actin) was used as an internal reference primer, and the gene-relative quantitative levels were estimated by the standard curve method [26].

### 2.8. Statistical Analysis

The statistical data were analyzed by SPSS (Version 20) and mean values were compared using one-way ANOVA and Tukey’s method. The values with *p* < 0.05 were considered statistically significant. The quadratic regression equation was used to analyze the dietary DL-Met and Met-Met requirements for *M. salmoides* using FCR indicators.

## 3. Results

### 3.1. Growth and Feed Utilization

**Figure 1:**
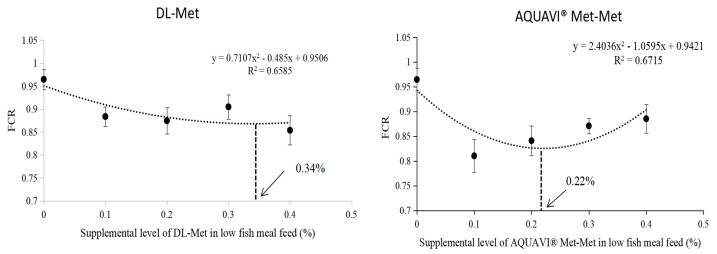
Quadratic regression equation analysis of feed conversion rate (FCR) in juvenile *M. salmoides* fed with different levels of DL-Met and Met-Met.

The results showed no significant difference in IW among the groups (*p* > 0.05; Table 6). Conversely, significantly decreased FW, WGR, and SGR and an increased feed conversion ratio (FCR) were observed in LF than in the HF group (*p* < 0.05). Moreover, the 0.40% DL-Met or 0.10% Met-Met groups showed similar values for FW, WGR, and SGR with the 40% FM group; however, only the 0.10% Met-Met group shows similar values with that of the 40% FM group for FCR (*p* < 0.05). In addition, the FI and SR of *M. salmoides* remained unaffected for all treatment groups (*p* > 0.05). The quadratic regression equation estimated the suitable dietary supplement of DL-Met and Met-Met as 0.34% or 0.22% of the diet, respectively, based on FCR (Figure 1).

### 3.2. The Whole Fish Composition and Amino Acid Composition

As shown in Table 7 and Table 8, no significant differences were observed in the whole fish and amino acid compositions among the groups (*p* > 0.05).

### 3.3. Intestinal Antioxidant Parameters

**Figure 2:**
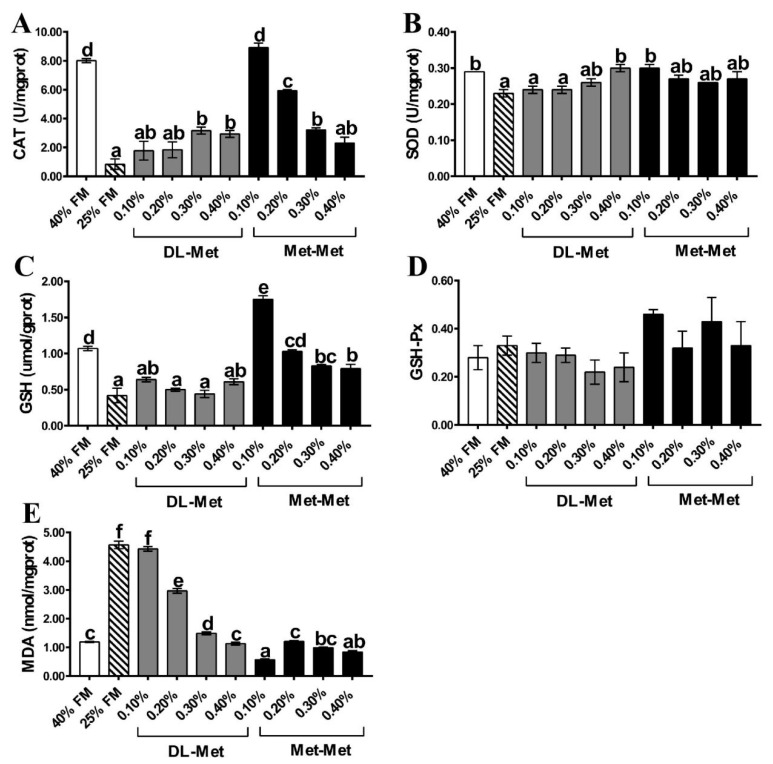
Activity of antioxidant-related parameters in the intestine of *M. salmoides*. Catalase (CAT) (**A**), superoxide dismutase (SOD) (**B**), glutathione (GSH) (**C**), glutathione peroxidase (GSH-Px) (**D**), malondialdehyde (MDA) (**E**). The 40% FM group (HF) and 25% FM group (LF). Data are presented as mean ± standard error, with different letters indicating differences within the groups (*p* < 0.05).

As shown in Figure 2, CAT, SOD, and GSH contents were significantly decreased in the LF group; however, the corresponding MDA levels were increased in comparison to the HF group. Moreover, the 0.30–0.40% DL-Met or 0.10–0.30% Met-Met groups showed significantly increased CAT activities relative to the LF group. Additionally, no differences were reported between the low-FM group supplemented with the 0.10% Met-Met and HF group. Furthermore, supplementation with 0.40% of DL-Met or 0.10% of Met-Met in the LF group significantly increased SOD activity in comparison to the LF group, but no significant difference was observed among the 0.40% DL-Met, 0.10% Met-Met, and HF groups. Moreover, Met-Met supplementation significantly increased GSH levels, whereas DL-Met supplementation had no significant effect when compared with the LF group. Specifically, supplementation with 0.10% of Met-Met significantly increased GSH composition compared with the 40% FM group. Moreover, 0.20–0.40% DL-Met or 0.10–0.40% Met-Met significantly reduced MDA contents in comparison to the LF group. In addition, no significant difference was observed between the 0.40% DL-Met and the HF groups; however, 0.10% Met-Met significantly reduced MDA content. On the other hand, GSH-Px activity was consistent among all the groups (*p* > 0.05).

### 3.4. Intestinal Antioxidant-Related Gene Levels

**Figure 3:**
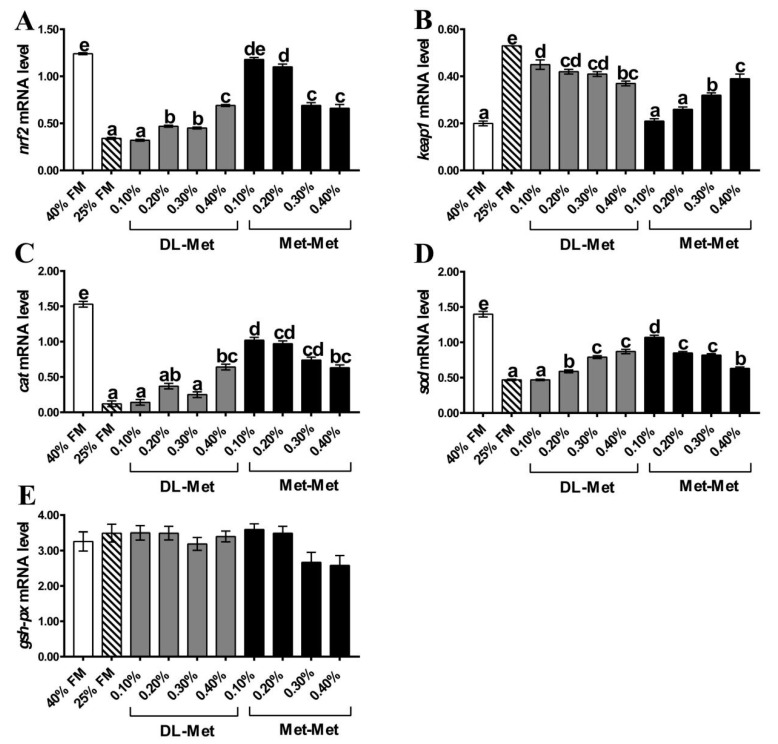
Expression of antioxidant-related genes in the intestine of *M. salmoides*. Nuclear factor erythroid 2-related factor 2 (*nrf2*) (**A**), kelch-like ECH-associated protein 1 (*keap1*) (**B**), catalase (*cat*) (**C**), superoxide dismutase (*sod*) (**D**), glutathione peroxidase (*gsh-px*) (**E**). The 40% FM group (HF) and 25% FM group (LF). Data are presented as mean ± standard error, with different letters indicating differences within the groups (*p* < 0.05).

Results demonstrated significantly downregulated expressions of the *nrf2*, *cat*, and *sod* genes and an upregulated expression of the *keap1* gene in the LF group compared with the HF group (*p* < 0.05; Figure 3). Moreover, the expressions of *nrf2* and *sod* genes were significantly upregulated by supplementation with 0.20–0.40% of DL-Met, whereas 0.40% of DL-Met upregulated *cat* gene expression and 0.10–0.40% of DL-Met significantly downregulated *keap1* gene expression when compared with the LF group (*p* < 0.05). The results of supplementation with 0.10–0.40% of Met-Met in the LF group showed significantly upregulated expressions of *nrf2*, *cat*, and *sod* genes and downregulated expression of the *keap1* gene relative to the LF group (*p* < 0.05). No difference was observed in the level of the *gsh-px* gene expression among the groups (*p* > 0.05).

### 3.5. Intestinal Inflammatory-Related Gene Levels

**Figure 4:**
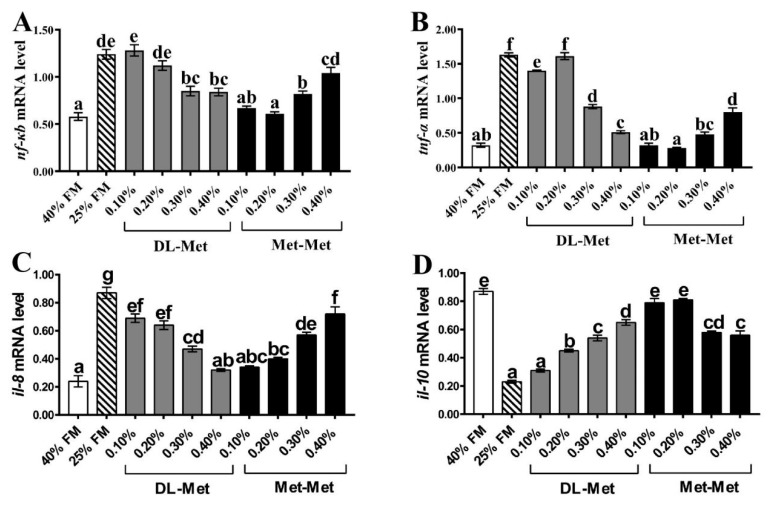
Expression of inflammatory-related genes in the intestine of *M. salmoides*. The nuclear factor κB (*nf-κb*) (**A**), tumor necrosis factor-alpha (*tnf-α*) (**B**), interleukin 8 (*il-8*) (**C**), interleukin 10 (*il-10*) (**D**). The 40% FM group (HF) and 25% FM group (LF). Data are presented as mean ± standard error, with different letters indicating differences within the groups (*p* < 0.05).

As shown in Figure 4, compared with the HF group, significantly upregulated expressions of *nf-κb*, *tnf-α*, and *il-8* genes and the downregulated expression of the *il-10* gene were observed in the LF group (*p* < 0.05). Furthermore, supplementation with 0.30–0.40% of DL-Met significantly decreased the expressions of the *nf-κb*, *tnf-α*, and *il-8* genes, respectively, whereas 0.20–0.40% of DL-Met significantly increased the expression of the *il-10* gene (*p* < 0.05). In addition, 0.10–0.30% of Met-Met significantly reduced the expression of the *nf-κb*, *tnf-α*, and *il-8* genes, while it increased the expression of the *il-10* gene relative to the LF group (*p* < 0.05).

### 3.6. Microbial Community Analysis in the Intestine

**Figure 5:**
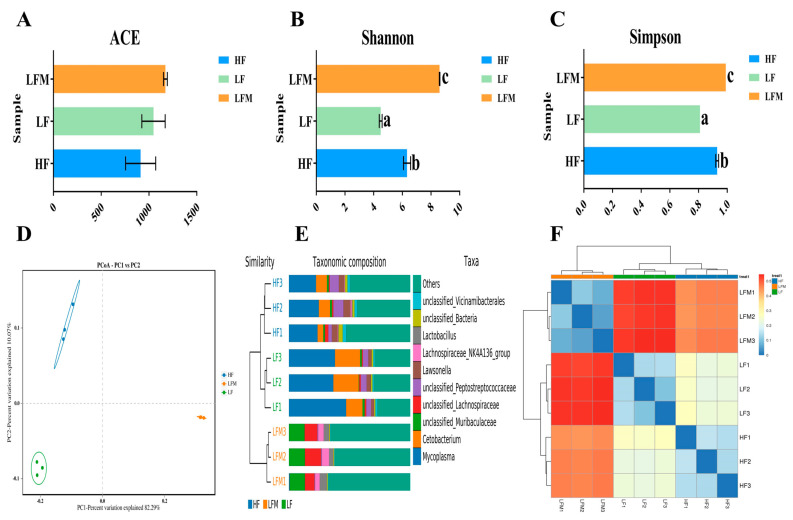
The intestinal tissue samples of three *M. salmoides* fed with 40% fishmeal (HF), 25% fishmeal (LF), and 25% fishmeal supplemented with 0.10% of Met-Met (LFM) were used for microbiological analysis. ACE and Simpson and Shannon indices of the HF, LF, and LFM groups were shown (**A**–**C**). The degree of proximity of the samples on the graph indicated the similarity level (**D**). The species diversity, abundance similarity, and dominant species of each sample were compared according to the intensity of each color (**E**). The color gradient from blue to red indicates the distance among samples from near to far (**F**). Data are presented as mean ± standard error, with different letters indicating differences within the groups (*p* < 0.05).

No difference was observed in the ACE index among the samples (*p* > 0.05; Figure 5A). Simpson and Shannon indices obtained the lowest and highest values in the LF and LFM groups, respectively (*p* < 0.05; Figure 5B,C). Principal coordinate analysis (PCoA) demonstrated differences in the intestinal microbiota of *M. salmoides* fed with three different diets (Figure 5D). The abundance histogram of each sample at the genus level was used to determine the similarity in microbial abundance among the samples. The species enrichment levels in three samples within the groups were similar, but they were different among the groups (Figure 5E). The results of the clustering Heatmap suggested that samples within the group were similar, while those among the groups were different (Figure 5F).

### 3.7. Intestinal Bacterial Community Phenotypes

**Figure 6:**
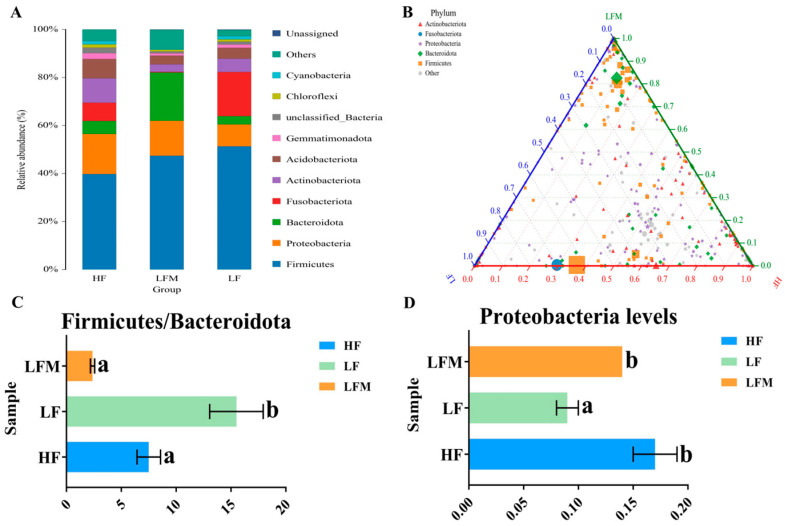

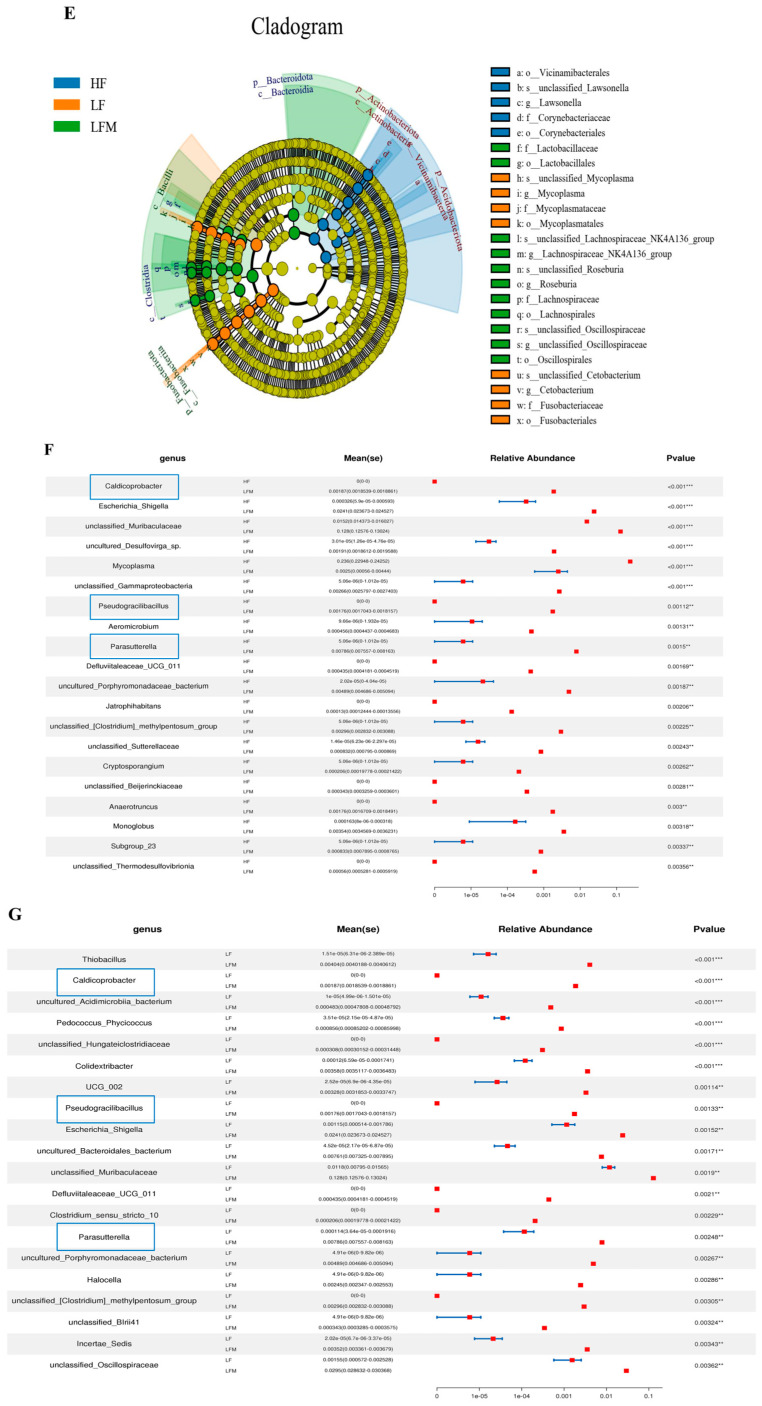
Each color represents one phylum level, and the color block length (bar chart) represents the relative abundance of the species, showing only the top ten phylum levels of abundance (**A**). Different shapes and points represent different phyla and genus, respectively, and the size of the points is the relative abundance of the genus. The three vertices represent three groups. The closer the point is to a vertex, the higher the abundance of the flora represented at that point in the sample represented at that vertex (**B**). Firmicutes/Bacteroidota represent the ratio of Firmicutes to Bacteroidota (**C**) and Proteobacteria levels (**D**) in three samples, with different letters indicating differences within the groups (*p* < 0.05); the circles radiating from the inside out represent taxonomic levels from phylum to species. Each small circle at a different classification level represents a classification at that level, and the diameter of the small circle is proportional to the relative abundance. The coloring rule uniformly imparts color to species with no significant differences as yellow, whereas species with differences are colored according to the highest abundance group where the species were located. Different colors represent different groups, and nodes with different colors represent microbial communities that occupy an important position in the groups represented by that color (**E**). The first and second columns provided information on species classification and grouping, respectively. The third and fourth columns listed the average abundance with standard error and relative abundance histograms of each group, respectively. *p* value < 0.05 was considered as a significant difference, ** *p* < 0.001, *** *p* < 0.0001, and the blue boxs represents the significantly increased bacterial genera in the LFM group compared to the HF and LF groups, respectively (**F**,**G**).

The horizontal community structure of the intestinal microbiota in three groups is shown in Figure 6A. At the phylum level, the abundance of Proteobacteria, Bacteroidota, Actinobacteriota, and Acidobacteriota decreased in the LF relative to the HF group, while that of the Firmicutes and Fusobacteriota increased. Simultaneously, the abundance of Proteobacteria and Bacteroidota increased in the LFM compared with the LF group but decreased for Fusobacteriota. The ternary plot could visually reveal the abundance of different species in the samples. As shown in Figure 6B, the species enrichment level was higher in the LFM, followed by HF, and it was lowest in the LF group. LEfSe analysis of the evolutionary branching map showed the differential bacterial taxa in the intestine of *M. salmoides* fed with HF, LF, and LFM diets. In addition, the ratio of Firmicutes to Bacteroidota in the LF group was significantly higher than that in the HF and LFM groups (*p* < 0.05; Figure 6C). Moreover, Proteobacteria were markedly lower in the LF group (*p* < 0.05; Figure 6D). In the cladogram, compared with the HF and LF groups, *Roseburia*, *Lachnospiraceae_NK4A136_group*, and *unclassified_Oscillospiraceae* were unique within the LFM group (Figure 6E). The differences in genus abundance between the two groups were analyzed by Metastats. Figure 6F,G show the comparison among genera of the LFM group with HF and LF groups, respectively.

## 4. Discussion

Fishmeal is widely used in aquatic animal feed due to its balanced amino acids [30]. Compared to fishmeal, soy protein is known for its low levels of methionine [31]. In the present study, the content of methionine in soybean meal is the lowest among several main ingredients in feed (as shown in the Supplementary Materials). In addition, compared with the plant protein source used in this study, the essential amino acid content of poultry meal is more similar to that of fishmeal, except for lysine and methionine (as shown in the Supplementary Materials), which are also the main limiting amino acids in poultry by-products [32]. Therefore, in the present study, methionine was supplemented with a low-fishmeal diet to explore its application effect in *M. salmoides* feed. Peptides have attracted widespread attention due to their unique transport mechanisms, leading to faster and more effective absorption rates than free-form crystalline amino acids in the intestines [21,33,34]. A study on DL-Methionine (DL-Met) and Met-Met showed that dietary Met-Met supplementation was more effective than DL-Met in *L. vannamei* [35]. Our results showed that growth-related indicators and feed utilization were decreased by reducing FM from 40 to 25%. However, compared to 25% FM, the supplementation of 25% FM with 0.40% DL-Met or 0.10% Met-Met significantly improved FW, WGR, and SGR. These results suggested the role of DL-Met or Met-Met supplementation in alleviating the negative effects of low-FM diets. Previous studies showed that DL-Met or Met-Met could promote the growth of larvae, juvenile Red Sea Bream [21], and *L. vannamei* [35]. According to the quadratic regression analysis of the FCR index, 0.34% DL-Met or 0.22% Met-Met added to LF meal resulted in similar growth and feed utilization by *M. salmoides* as those feeding on high FM. Therefore, it can be concluded that the *M. salmoides* diet with low FM required a higher level of DL-Met supplementation than that of Met-Met to induce desirable growth. In other words, *M. salmoides* shows better utilization of Met-Met than DL-Met. In addition, Xie et al. reported that an improved growth performance of *L. vanname* could be achieved by adding 0.10% of Met-Met or 0.30% of DL-Met to a low-FM diet [35], which further validated our results. Moreover, in the present study, no significant difference was observed in the whole-body and amino acid composition of *M. salmoides* among the groups. The results were similar to those reported in the previous studies on *L. vannamei* [36] and Red Sea Bream [21].

Dietary low-FM contents lead to oxidative stress in *M. salmoides* [37], which is relieved by antioxidant enzymes and related genes mainly through the Nrf2 signaling pathway [38,39]. Oxidative stress results from the overproduction of reactive oxygen species (ROS), which are removed by the action of antioxidant enzymes (such as SOD, GSH-Px, and CAT) [40]. One of the important indicators of oxidative damage in the body is the increased level of MDA [16]. In the present study, relative to the HF diet, feeding with the LF diet significantly reduced the intestinal CAT, SOD, and GSH levels, whereas the MDA content was increased. This suggests that consuming a low-FM diet could reduce the antioxidant ability of the intestines of *M. salmoides,* resulting in oxidative damage. Furthermore, in comparison to the LF group, CAT, SOD, and GSH levels in the intestines of *M. salmoides* increased significantly when DL-Met or Met-Met were added to LF diets. Specifically, 0.20–0.40% of DL-Met or 0.10–0.40% of Met-Met significantly reduced MDA content relative to the LF group. Previous studies on Nile tilapia [20] and *L. vannamei* [23] reported that 0.20% and 0.10–0.15% of Met-Met increased the antioxidant capacity, respectively. Ji et al. [23] also reported that 0.10–0.25% of Met-Met significantly reduced the MDA content in *L. vannamei*. Collectively, these results indicated that supplementing with DL-Met or Met-Met could effectively alleviate the intestinal oxidative damage caused by a low-FM diet. Particularly, 0.10% of Met-Met increased GSH and decreased MDA content in comparison to the HF group, suggesting a more effective role of Met-Met than that of DL-Met.

The nrf2-mediated gene expression of antioxidant enzymes reduces external oxidative stress [41]. In the present study, relative to the HF group, the LF diet significantly reduced the expressions of *nrf2*, *cat*, and *sod* genes, whereas the *keap1* gene expression levels were significantly increased. According to a study, dietary low FM could reduce the antioxidant capacity in juvenile golden pompano (*Trachinotus ovatus*) by decreasing the *nrf2* expression level in the nrf2 signaling pathway, increasing the *keap1* expression level, thereby inhibiting the expression of oxidative stress-related genes, such as *sod*, *cat*, and *gsh-px* [42]. Similarly, a low-FM diet for *M. salmoides* could downregulate the intestinal antioxidant capacity through the nrf2-mediated expression of antioxidant enzymes and related genes. Compared with the LF group, 0.20–0.40% of DL-Met supplementation significantly upregulated *nrf2* and *sod* genes expression and downregulated *keap1* gene expression, whereas 0.10–0.40% of Met-Met the upregulated *nrf2*, *cat*, and *sod* and downregulated the *keap1* gene expression in the present study. Conclusively, DL-Met or Met-Met improved the antioxidant capacity of *M. salmoides* by regulating the activity of related enzymes and genes of the Nrf2 signaling pathway.

The nuclear factor (nf-κb) can regulate inflammation through cytokines. As a transcription factor, it regulates the expression of pro-inflammatory genes, including *tnf-α* and *il-8*, and anti-inflammatory genes, such as *il-10* [43,44]. In the present study, significantly upregulated levels of the *nf-κb*, *tnf-α*, and *il-8* genes and downregulated levels of *il-10* were induced by the LF diet relative to the HF group, which is consistent with the results of a previous study [45]. In addition, our results showed that in comparison to the LF group, supplementation with DL-Met or Met-Met significantly down- and upregulated the pro- and anti-inflammatory genes, respectively, in the intestine of *M. salmoides*. The above results indicated that consumption of a low-FM diet could lead to intestinal inflammation in *M. salmoides*, and DL-Met or Met-Met supplementation could alleviate the inflammatory response.

The intestinal microbiome is crucial for human health and is substantially influenced by diet [46]. Similarly, in fish, intestinal microbiota plays an important role in nutrition, immunity, and resistance to invading pathogens, and diet greatly influences its composition [47]. According to a study on Nile tilapia, the addition of Met-Met to fish feed could influence the composition of intestinal microbiota [20]. The core microbiota are closely related to the host genotype and remain unaffected by the external environment [48,49]. In the present study, the intestinal tissue samples of *M. salmoides* fed with the HF, LF, and LFM diets were used for microbiological analysis. The ACE and Simpson and Shannon indices in α-diversity are commonly used to evaluate the species richness and diversity. Our results did not show a significant difference in ACE among the groups, and the lowest values of the Simpson and Shannon index were observed in the LF group, which were significantly increased by Met-Met supplementation. This suggests the potential of Met-Met in enhancing the diversity of intestinal microbiota. PCoA, UPGMA, and sample clustering heatmaps for β-diversity analysis revealed that the microorganisms in the three random samples within the groups were similar; however, there were significant differences in the microbiota among the groups.

Nevertheless, an imbalanced Firmicutes-to-Bacteroidota ratio could lead to pathogenic invasion [50]. In the present study, the Firmicutes-to-Bacteroidota ratio of the LF group was significantly higher than that of the HF and LFM groups. This suggested that the low-FM diet may lead to an imbalanced Firmicutes-to-Bacteroidota ratio in the intestine of *M. salmoides*, which was more likely to promote pathogenic invasion, thereby reducing the immunity of fish. However, the supplementation of Met-Met in a low-FM diet could improve intestinal immunity by regulating the Firmicutes-to-Bacteroidota ratio. Furthermore, Proteobacteria is the core intestinal microbiota of fish [51,52]. Previous studies on the intestinal tissue samples of grass carp (*Ctenopharyngodon idella*) revealed that Proteobacteria is positively correlated with the anti-inflammatory factor *tgf-β*, suggesting its role in improving the anti-inflammatory ability of fish [53]. Our results showed significantly lower levels of Proteobacteria in the LM group relative to the HF and LFM groups. Moreover, the upregulation of pro-inflammatory factors and downregulation of anti-inflammatory factors might be induced by the imbalanced Firmicutes-to-Bacteroidota ratio or reduced levels of Proteobacteria in a low-FM diet. However, the relevant regulatory mechanisms of bacterial abundance and inflammatory factors need further investigation. The results also demonstrated that Met-Met may improve intestinal health by increasing the abundance of beneficial bacteria. Additionally, the results of the ternary plot showed that Met-Met changed the bacterial diversity, whereas the LEfSe analysis revealed that the *unique Roseburia* [54,55], *Lachnospiraceae_NK4A136_group* [56], and *unclassified_Oscillospiraceae* [57] in the LFM group were beneficial bacteria. *Roseburia* was negatively correlated with MDA level and positively correlated with antioxidant enzymes, including SOD in the gut of mice [58]. Moreover, it plays an anti-inflammatory role in alleviating colitis pathology, suggesting its potential anti-inflammatory effects [59]. Similarly, *Lachnospiraceae_NK4A136_group* found in the gut of mice was positively correlated with anti-inflammatory genes and negatively correlated with the pro-inflammatory and oxidative stress factors [60]. Therefore, it can be concluded that changes in the abundance of *Roseburia* and *Lachnospiraceae_NK4A136_group* may have resulted from Met-Met supplementation, thereby enhancing the intestinal oxidation and anti-inflammatory capacity of *M. salmoides*. Notably, *unclassified Oscillospiraceae* produces butyrate after fermentation in the gut, which helps in the utilization of dietary fiber [61]. The differences in genus abundance between the two groups were analyzed by Metastats. In the present study, compared with the LF and HF groups, the abundance of probiotic bacteria, such as *Caldicoprobacter*, *Pseudogracilibacillus*, and *Parasutterella*, was increased in the LFM group. Moreover, *Caldicoprobacter* could ferment complex carbohydrates in the intestine to produce lactic acid, thereby promoting the production of short-chain fatty acids to maintain intestinal health [62,63]. *Pseudogracilibacilluse* and *Parasutterella* are considered beneficial bacteria [64,65]. In addition, anti-inflammatory effects may be exerted by the metabolites (7-ketodeoxycholic acid and haloperidol glucuronide) produced by *Parasutterella* [66]. This implies that the supplementation of Met-Met in a low-FM diet could improve the composition and abundance of beneficial bacteria in the gut, thereby improving intestinal health.

## 5. Conclusions

An FM reduction from 40 to 25% could affect the growth performance and feed utilization, whereas the supplementation with DL-Met or Met-Met improved these parameters. In addition, Met-Met could enhance the antioxidant and anti-inflammatory capacity and effectively regulate the abundance of dominant bacteria to promote the intestinal health of *M. salmoides*. Moreover, the quadratic regression analysis evaluated the suitable dietary supplementation of DL-Met or Met-Met as 0.34% or 0.22% of the diet, respectively, based on FCR. Finally, relative to DL-Met, Met-Met demonstrated a better utilization rate, and results demonstrated that the effects produced by 0.09% of Met-Met were equivalent to those produced by 0.36% of DL-Met supplementation.

## Figures and Tables

**Table 1:** The key indicators of water quality monitoring during the culture cycle.

Water Environmental Factors	Range
Temperature	15–20 °C
Dissolved oxygen	>6 mg/L
Ammonia nitrogen content	<0.1 mg/L
pH	6.8–7.5

**Table 2:** Ingredient and proximate analysis of the experimental diets.

Item	Diets									
	40% FM	25% FM	0.10% DL-Met	0.20% DL-Met	0.30% DL-Met	0.40% DL-Met	0.10% Met-Met	0.20% Met-Met	0.30% Met-Met	0.40% Met-Met
Fishmeal ^1^	40	25	25	25	25	25	25	25	25	25
Poultry meal ^1^	1	15	15	15	15	15	15	15	15	15
Blood cell protein ^1^	3	3	3	3	3	3	3	3	3	3
Soybean meal ^1^	18	18	18	18	18	18	18	18	18	18
Corn gluten meal ^1^	2	2	2	2	2	2	2	2	2	2
Soy protein concentrate ^1^	7	7	7	7	7	7	7	7	7	7
Shrimp paste	2	2	2	2	2	2	2	2	2	2
Rice bran	4.98	3.65	3.53	3.4	3.28	3.15	3.53	3.4	3.28	3.15
Wheat	6	6	6	6	6	6	6	6	6	6
Tapioca	4	4	4	4	4	4	4	4	4	4
Fish oil	5.87	6.02	6.04	6.06	6.08	6.1	6.04	6.06	6.08	6.1
Soybean oil	2	2	2	2	2	2	2	2	2	2
Monocalcium phosphate	2	3.66	3.66	3.66	3.66	3.67	3.66	3.66	3.66	3.67
Choline chloride	0.3	0.3	0.3	0.3	0.3	0.3	0.3	0.3	0.3	0.3
L-carnitine hydrochloride	0.3	0.3	0.3	0.3	0.3	0.3	0.3	0.3	0.3	0.3
L-ascorbate-2-phosphate ester	0.05	0.05	0.05	0.05	0.05	0.05	0.05	0.05	0.05	0.05
Vitamin premix feed for carnivorous fish ^2^	0.5	0.5	0.5	0.5	0.5	0.5	0.5	0.5	0.5	0.5
Trace mineral premix for carnivorous fish ^2^	1	1	1	1	1	1	1	1	1	1
L-Lysine ^3^	0	0.29	0.29	0.3	0.3	0.3	0.29	0.3	0.3	0.3
DL-Met	0	0	0.1	0.2	0.3	0.4	0	0	0	0
Met-Met	0	0	0	0	0	0	0.1	0.2	0.3	0.4
L-threonine ^3^	0	0.23	0.23	0.23	0.23	0.23	0.23	0.23	0.23	0.23
Proximate analysis (% dry basis)										
Protein	47.48	47.28	47.4	47.22	47.51	47.55	47.3	47.45	47.37	47.27
Lipid	12.04	11.95	12.1	12.12	12.13	12.09	12.12	12.02	12.18	12
Gross energy (KJ/g)	20.11	19.77	20	19.99	20.05	19.69	19.89	19.68	19.53	19.65

Note: ^1^ Fishmeal and other major protein sources were purchased from Wuxi Tongwei feedstuffs Co., Ltd. (Wuxi, China); ^2^ Vitamin premix and trace mineral premix were obtained from HANOVE Animal Health Products (IU, mg/kg of premix). Vitamin premix (IU or mg/kg of premix): vitamin A, 800,000 IU; vitamin D3, 250,000 IU; vitamin E, 4500 IU; vitamin K3, 600 mg; thiamin, 800 mg; ribofavin, 800 mg; calcium pantothenate, 2000 mg; pyridoxine HCl, 2500 mg; cyanocobalamin, 8 mg; biotin, 16 mg; folic acid, 400 mg; niacin, 2800 mg; inositol, 10,000 mg; vitamin C, 10,000 mg. Mineral premix (g/kg of premix): magnesium sulfate, 15 g; ferrous sulfate, 30 g; zinc sulfate, 13.5 g; cupric sulfate, 0.8 g; manganese sulfate, 6 g; zeolite was used as a carrier. ^3^ L-Lysine and L-threonine obtained from Feeer Co., Ltd. (Shanghai, China).

**Table 3:** Amino acid content in diets and Met-Met and DL-Met levels after supplementation.

Item	Diets									
	40% FM	25% FM	0.10% DL-Met	0.20% DL-Met	0.30% DL-Met	0.40% DL-Met	0.10% Met-Met	0.20% Met-Met	0.30% Met-Met	0.40% Met-Met
Essential amino acid										
Met	1.03	0.94	1.04	1.13	1.23	1.31	1.10	1.15	1.23	1.35
Lys	3.17	3.13	3.12	3.12	3.13	3.15	3.20	3.14	3.07	3.13
Thr	1.80	1.98	1.98	1.99	1.98	2.00	2.05	1.99	1.94	1.98
Arg	2.68	2.83	2.92	2.91	2.89	2.89	2.90	2.87	2.80	2.92
ILe	1.89	1.99	1.98	1.98	1.99	2.01	2.03	1.99	1.95	1.98
Leu	3.62	3.61	3.60	3.62	3.60	3.65	3.71	3.62	3.56	3.62
Val	2.34	2.35	2.32	2.31	2.29	2.32	2.39	2.32	2.28	2.33
His	1.41	1.29	1.29	1.29	1.28	1.30	1.31	1.29	1.26	1.29
Phe	2.14	2.12	2.10	2.11	2.09	2.13	2.15	2.11	2.07	2.11
Nonessential amino acid										
Gly	2.48	2.66	2.64	2.63	2.64	2.67	2.74	2.65	2.59	2.66
Ser	1.91	2.00	2.02	2.03	2.01	2.03	2.08	2.01	1.97	2.02
Pro	2.08	2.19	2.23	2.20	2.21	2.21	2.26	2.24	2.18	2.31
Ala	2.68	2.62	2.61	2.62	2.62	2.64	2.72	2.63	2.58	2.63
Asp	4.49	4.37	4.37	4.37	4.38	4.41	4.48	4.39	4.28	4.37
Glu	6.61	6.58	6.53	6.56	6.55	6.62	6.72	6.58	6.45	6.61
Cys	0.50	0.54	0.53	0.53	0.53	0.54	0.56	0.53	0.53	0.53
Met + Cys	1.53	1.48	1.57	1.66	1.76	1.85	1.65	1.68	1.76	1.88
Other parameters (after supplementation)										
DL-Met	0.01	0.02	0.10	0.18	0.27	0.36	0.02	0.02	0.02	0.03
Met-Met	<0.01	<0.01	<0.01	<0.01	<0.01	<0.01	0.09	0.17	0.26	0.37

Note: Methionine (Met), lysine (Lys), threonine (Thr), arginine (Arg), isoleucine (ILe), leucine (Leu), valine (Val), histidine (His), phenylalanine (Phe), glycine (Gly), serine (Ser), proline (Pro), alanine (Ala), aspartic acid (Asp), glutamic acid (Glu), serine (Cys).

**Table 4:** Experimental assay methods with details of manufacturers or assay kits utilized for sample analyses.

Items	Methods	Assay Kits/Manufacturer
GSH	The intestine tissues of the *M. salmoides* fed with different diets were crushed and mixed with ice-cold normal saline; afterwards, centrifuging according to the instructions in the manual to obtain the supernatant was used to detect antioxidant parameters according to the manufacturer’s instructions.	The intestinal antioxidant parameters were detected using the kits purchased from Nanjing Jiancheng Bioengineering Institute (Nanjing, China).
GSH-Px		
SOD		
CAT		
MDA		
Crude protein	The nutritional components of experimental fish and feed were tested by AOAC [24].	Detected by automatic Kjeldahl nitrogen analyzer (K9840) (Hanon Advanced Technology Group Co., Ltd., Jinan, China).
Crude fat		Extracted according to the soxhlet extraction method.
Crude ash		Detected by incineration in a muffle furnace at 550 °C for 24 h (XL-2A) (Hangzhou Zhuochi Instrument Co., Ltd., Hangzhou, China).
Moisture		Detected by an oven (105 °C) (Shanghai Yiheng Scientific Instrument Co., Ltd., Shanghai, China).
Gross energy		Measured with an oxygen bomb calorimeter (C6000, IKA) (Staufen, Germany).
Amino acid		The amino acid contents, except that of tryptophan, were measured by an amino acid analyzer (SYKAM S-433D, Germany SYKAM Instruments Co., Ltd., Eresing, Germany). The level of tryptophan was measured on the instrument after alkaline hydrolysis (5 mol/L NaOH, 110 °C, 20 h).

Note: glutathion (GSH), glutathione peroxidase (GSH-Px), superoxide dismutase (SOD), catalase (CAT), malondialdehyde (MDA).

**Table 5:** Primer sequences for qPCR.

Gene Name		Sequence	Accession Number/Reference
*nrf2*	F	CCACACGTGACTCTGATTTCTC	Gene ID: 119904119 (Transcriptome data)
	R	TCCTCCATGACCTTGAAGCAT	
*sod*	F	CCACCAGAGGTCTCACAGCA	[27]
	R	CCACTGAACCGAAGAAGGACT	
*cat*	F	CTATGGCTCTCACACCTTC	MK614708.1
	R	TCCTCTACTGGCAGATTCT	
*gsh-px*	F	ATGGCTCTCATGACTGATCCAAA	MK614713.1
	R	GACCAACCAGGAACTTCTCAAA	
*keap1*	F	GCACCTAACCGTGGAACTCAA	[28]
	R	CCAGTTTTAGCCAGTCATTGTTCC	
*nf-κb*	F	AGAAGACGACTCGGGGATGA	[27]
	R	GCTTCTGCAGGTTCTGGTCT	
*tnf-α*	F	CTTCGTCTACAGCCAGGCATCG	[29]
	R	TTTGGCACACCGACCTCACC	
*il-8*	F	GAGGGTACATGTCTGGGGGA	XM_038713529.1
	R	CCTTGAAGGTTTGTTCTTCATCGT	
*il-10*	F	CGGCACAGAAATCCCAGAGC	[29]
	R	CAGCAGGCTCACAAAATAAACATCT	
*β-actin*	F	ATGCAGAAGGAGATCACAGCCT	AF253319.1
	R	AGTATTTACGCTCAGGTGGGG	

Note: nuclear factor erythroid 2-related factor 2 (*nrf2*), superoxide dismutase (*sod*), catalase (*cat*), glutathione peroxidase (*gsh-px*), kelch-like ECH-associated protein 1 (*keap1*), the nuclear factor κB (*nf-κb*), tumor necrosis factor-alpha (*tnf-α*), interleukin 8 (*il-8*), interleukin 10 (*il-10*), beta-actin (*β-actin*).

**Table 6:** Effects of different levels of Met-Met and DL-Met supplementation on the growth and feed utilization in juvenile *M. salmoides*.

Item	Diets ^1^									
	40% FM	25% FM	0.10% DL-Met	0.20% DL-Met	0.30% DL-Met	0.40% DL-Met	0.10% Met-Met	0.20% Met-Met	0.30% Met-Met	0.40% Met-Met
IW ^2^, g	16.38 ± 0.03	16.38 ± 0.03	16.39 ± 0.04	16.39 ± 0.03	16.38 ± 0.03	16.41 ± 0.03	16.39 ± 0.02	16.41 ± 0.03	16.40 ± 0.02	16.39 ± 0.02
FW ^3^, g	60.16 ± 0.73 ^b^	52.34 ± 0.79 ^a^	55.84 ± 1.12 ^ab^	55.94 ± 1.08 ^ab^	56.57 ± 1.73 ^ab^	58.63 ± 1.80 ^b^	58.96 ± 1.26 ^b^	58.15 ± 1.00 ^ab^	58.08 ± 1.48 ^ab^	54.8 ± 1.10 ^ab^
WGR ^4^, %	267.24 ± 4.46 ^b^	219.51 ± 4.60 ^a^	240.79 ± 6.99 ^ab^	241.39 ± 6.51 ^ab^	245.24 ± 10.13 ^ab^	257.40 ± 11.33 ^b^	259.675 ± 8.06 ^b^	254.42 ± 5.84 ^ab^	254.20 ± 9.20 ^ab^	234.40 ± 6.83 ^ab^
SGR ^5^, %/d	1.86 ± 0.02 ^b^	1.66 ± 0.02 ^a^	1.75 ± 0.03 ^ab^	1.75 ± 0.03 ^ab^	1.77 ± 0.04 ^ab^	1.82 ± 0.05^b^	1.83 ± 0.03 ^b^	1.81 ± 0.02 ^ab^	1.81 ± 0.04 ^ab^	1.72 ± 0.03 ^ab^
FCR ^6^	0.79 ± 0.01 ^a^	0.96 ± 0.02 ^b^	0.88 ± 0.02 ^ab^	0.87 ± 0.02 ^ab^	0.91 ± 0.03 ^ab^	0.85 ± 0.03 ^ab^	0.81 ± 0.03 ^a^	0.84 ± 0.03 ^ab^	0.87 ± 0.02 ^ab^	0.89 ± 0.03 ^ab^
FI ^7^, g/fish/d	0.32 ± 0.01	0.36 ± 0.01	0.34 ± 0.01	0.34 ± 0.01	0.35 ± 0.01	0.34 ± 0.01	0.33 ± 0.01	0.34 ± 0.01	0.34 ± 0.01	0.34 ± 0.01
SR ^8^, %	100	99.00 ± 1.00	100	100	98.00 ± 2.00	99.00 ± 1.00	100	100	98.00 ± 1.15	100

^1^ The 40% FM group (HF) and 25% FM group (LF). Data are presented as mean ± standard error, with different letters indicating differences within the groups (*p* < 0.05). ^2^ Initial average body weight. ^3^ Final average body weight. ^4^ Weight gain rate (WGR) (%) = 100 × (final body average weight (g) − initial body average weight (g))/initial weight (g). ^5^ Specific growth rate (SGR) (%/d) = 100 × ((ln (final body average weight (g)) − ln (initial body average weight (g)))/days). ^6^ Feed conversion ratio (FCR) = dry feed fed (g)/wet weight gain (g). ^7^ Feed intake (FI) (g fish^−1^ d^−1^) = dry feed fed (g)/((final body weight) (g) + initial body weight (g))/2 × days. ^8^ Survival rate (SR) (%) = 100 × (final number of fish/initial number of fish).

**Table 7:** Effects of Met-Met and DL-Met supplementation on whole body composition in juvenile *M. salmoides*.

Item	Diets									
	40% FM	25% FM	0.10% DL-Met	0.20% DL-Met	0.30% DL-Met	0.40% DL-Met	0.10% Met-Met	0.20% Met-Met	0.30% Met-Met	0.40% Met-Met
Moisture, %	73.65 ± 0.10	73.62 ± 0.04	73.70 ± 0.37	73.64 ± 0.13	73.37 ± 0.35	73.74 ± 0.10	73.70 ± 0.19	74.56 ± 0.67	73.38 ± 0.49	73.61 ± 0.10
Lipid, %	5.66 ± 0.15	5.55 ± 0.11	5.24 ± 0.20	5.40 ± 0.08	5.40 ± 0.23	5.49 ± 0.09	5.57 ± 0.08	5.21 ± 0.23	5.34 ± 0.19	5.48 ± 0.14
Ash, %	3.94 ± 0.06	3.86 ± 0.10	4.20 ± 0.08	4.25 ± 0.12	4.21 ± 0.18	4.25 ± 0.04	3.89 ± 0.21	4.13 ± 0.02	4.11 ± 0.10	4.21 ± 0.03
Protein, %	17.31 ± 0.02	17.26 ± 0.12	17.24 ± 0.01	17.18 ± 0.09	17.04 ± 0.13	17.05 ± 0.10	17.06 ± 0.04	17.26 ± 0.02	17.16 ± 0.02	16.95 ± 0.18

Note: 40% FM group (HF) and 25% FM group (LF). Data are presented as mean ± standard error.

**Table 8:** Effects of Met-Met and DL-Met supplementation on amino acid composition of whole fish in juvenile *M. salmoides*.

Item	Diets									
	40% FM	25% FM	0.10% DL-Met	0.20% DL-Met	0.30% DL-Met	0.40% DL-Met	0.10% Met-Met	0.20% Met-Met	0.30% Met-Met	0.40% Met-Met
Essential amino acid										
Met	1.97 ± 0.26	2.02 ± 0.23	2.10 ± 0.22	2.11 ± 0.21	2.02 ± 0.22	2.07 ± 0.21	2.06 ± 0.21	2.86 ± 0.02	1.86 ± 0.03	1.86 ± 0.00
Lys	5.76 ± 0.87	5.92 ± 0.76	6.05 ± 0.75	6.10 ± 0.71	5.87 ± 0.75	6.05 ± 0.75	6.00 ± 0.71	5.36 ± 0.03	5.28 ± 0.11	5.29 ± 0.02
Thr	3.11 ± 0.32	3.19 ± 0.23	3.29 ± 0.24	3.34 ± 0.27	3.23 ± 0.27	3.29 ± 0.22	3.27 ± 0.22	3.05 ± 0.02	3.01 ± 0.05	3.05 ± 0.01
Trp	0.69 ± 0.01	0.63 ± 0.02	0.67 ± 0.01	0.67 ± 0.02	0.65 ± 0.02	0.66 ± 0.01	0.65 ± 0.02	0.68 ± 0.00	0.64 ± 0.02	0.68 ± 0.00
Arg	4.31 ± 0.36	4.48 ± 0.23	4.53 ± 0.26	4.56 ± 0.24	4.44 ± 0.23	4.53 ± 0.24	4.52 ± 0.23	4.32 ± 0.02	4.30 ± 0.05	4.38 ± 0.02
ILe	3.03 ± 0.39	3.19 ± 0.38	3.33 ± 0.36	3.36 ± 0.35	3.26 ± 0.37	3.35 ± 0.36	3.33 ± 0.35	2.99 ± 0.02	2.96 ± 0.08	2.96 ± 0.02
Leu	5.24 ± 0.70	5.41 ± 0.59	5.54 ± 0.60	5.60 ± 0.57	5.44 ± 0.61	5.59 ± 0.57	5.56 ± 0.57	5.02 ± 0.02	4.92 ± 0.10	4.95 ± 0.03
Val	3.39 ± 0.36	3.59 ± 0.32	3.72 ± 0.31	3.77 ± 0.31	3.66 ± 0.31	3.76 ± 0.28	3.75 ± 0.29	3.47 ± 0.01	3.40 ± 0.08	3.44 ± 0.01
His	1.64 ± 0.18	1.72 ± 0.15	1.74 ± 0.13	1.79 ± 0.12	1.71 ± 0.14	1.76 ± 0.15	1.73 ± 0.13	1.59 ± 0.01	1.60 ± 0.03	1.60 ± 0.01
Phe	3.24 ± 0.33	3.35 ± 0.26	3.43 ± 0.28	3.48 ± 0.26	3.31 ± 0.26	3.36 ± 0.26	3.40 ± 0.27	3.28 ± 0.04	3.21 ± 0.06	3.22 ± 0.04
Nonessential amino acid										
Gly	5.36 ± 0.27	5.77 ± 0.40	5.86 ± 0.38	5.68 ± 0.36	5.78 ± 0.39	5.76 ± 0.40	5.68 ± 0.44	6.02 ± 0.04	6.02 ± 0.03	6.32 ± 0.10
Ser	2.87 ± 0.26	2.93 ± 0.16	2.95 ± 0.17	2.97 ± 0.18	2.92 ± 0.19	2.97 ± 0.16	2.93 ± 0.17	2.80 ± 0.02	2.75 ± 0.05	2.81 ± 0.03
Pro	3.50 ± 0.16	3.79 ± 0.19	3.82 ± 0.19	3.74 ± 0.20	3.76 ± 0.19	3.83 ± 0.22	3.74 ± 0.21	3.90 ± 0.02	3.81 ± 0.03	4.03 ± 0.03
Ala	4.75 ± 0.31	4.94 ± 0.14	5.04 ± 0.15	5.03 ± 0.15	4.95 ± 0.14	5.06 ± 0.15	5.00 ± 0.15	4.85 ± 0.01	4.79 ± 0.05	4.93 ± 0.03
Asp	9.92 ± 1.12	10.31 ± 0.89	10.53 ± 0.90	10.58 ± 0.86	10.32 ± 0.88	10.51 ± 0.85	10.51 ± 0.84	9.72 ± 0.06	9.54 ± 0.16	9.63 ± 0.07
Glu	9.92 ± 1.12	10.31 ± 0.89	10.53 ± 0.90	10.58 ± 0.86	10.32 ± 0.88	10.51 ± 0.85	10.51 ± 0.84	9.72 ± 0.06	9.54 ± 0.16	9.63 ± 0.07
Cys	0.64 ± 0.07	0.67 ± 0.07	0.67 ± 0.05	0.67 ± 0.06	0.62 ± 0.05	0.60 ± 0.06	0.60 ± 0.06	0.56 ± 0.01	0.56 ± 0.01	0.57 ± 0.00
Met+ Cys	2.61 ± 0.33	2.69 ± 0.29	2.77 ± 0.28	2.78 ± 0.27	2.65 ± 0.26	2.67 ± 0.27	2.66 ± 0.26	2.42 ± 0.03	2.42 ± 0.03	2.43 ± 0.01

Note: Data are presented as mean ± standard error. Methionine (Met), lysine (Lys), threonine (Thr), tryptophan (Trp), arginine (Arg), isoleucine (ILe), leucine (Leu), valine (Val), histidine (His), phenylalanine (Phe), glycine (Gly), serine (Ser), proline (Pro), alanine (Ala), aspartic acid (Asp), glutamic acid (Glu), serine (Cys).

## Data Availability

Data are contained within the article.

## Supplementary Materials

The following supporting information can be downloaded at: https://www.mdpi.com/article/10.3390/antiox13030359/s1, Table S1. The main ingredients’ chemical content and amino acid content.
