# Supplementary material for: DL-Methionyl–DL-Methionine/DL-Methionine Supplementation Alleviated the Adverse Effects of Dietary Low Fishmeal Levels on Growth and Intestinal Health of Micropterus salmoides
Source: Antioxidants (Basel). 2024 Mar 18;13(3):359. doi: 10.3390/antiox13030359 (PMC10967736; doi:10.3390/antiox13030359)
Supplement: Supplementary file 1 [file antioxidants-13-00359-s001.zip › antioxidants-2894681-supplementary.pdf]

Supplementary material

Table S1. The main ingredients' chemical content and amino acid content.

**Nutrient content of wet matter (%)**	**Ingredients**				
	Fish meal	Poultry meal	Soybean meal	Corn gluten meal	Soy protein concentrate
			Essential amino acid		
Arg	3.49	4.20	3.51	1.80	4.65
His	1.95	1.29	1.25	1.27	1.71
ILe	2.60	2.62	2.19	2.30	2.96
Leu	4.59	4.72	3.71	9.62	5.00
Lys	4.93	3.88	3.01	0.94	4.10
Met	1.79	1.33	0.68	1.36	0.90
Phe	2.77	2.78	2.58	3.67	3.40
Thr	2.66	2.61	1.92	1.92	2.57
Val	3.06	3.21	2.30	2.66	3.08
			Nonessential amino acid		
Cys	0.64	1.00	0.70	1.09	0.90
Met+ Cys	2.43	2.33	1.38	2.45	1.80
Gly	3.94	5.28	2.05	1.59	2.71
Ser	2.38	3.28	2.41	2.99	3.25
Pro	2.76	4.08	2.61	5.80	3.54
Ala	3.99	3.78	2.10	5.05	2.77
Asp	5.83	5.32	5.59	3.48	7.45
Glu	8.18	8.41	8.77	12.73	11.64
			Main chemical content		
Dry matter	93.23	91.81	89.58	92.80	90.63
Crude protein	66.44	66.89	49.92	60.91	66.08
Crude fat	6.30	7.25	3.25	-	-
Gross energy (KJ/g)	23.70	27.90	24.20	28.30	24.80
